# Supplementary material for: Pulmonary Pharmacokinetic and Pharmacodynamic Evaluation of Ampicillin/Sulbactam Regimens for Pneumonia Caused by Various Bacteria, including Acinetobacter baumannii
Source: Antibiotics (Basel). 2023 Feb 2;12(2):303. doi: 10.3390/antibiotics12020303 (PMC9952633; doi:10.3390/antibiotics12020303)
Supplement: Supplementary file 1 [file antibiotics-12-00303-s001.zip › antibiotics-2175669-supplementary.pdf]

Table S1 Demographic information of literature data used in this study

	PK parameter in blood concentrations<br>・Onita T et al. [19])	Calculation of KP_{lung}<br>・Frank U et al. [16]	Physiological parameter<br>・Langdon G et al. [24]<br>・Brown RP et al. [25]
**The number of the subjects**	N = 44 (All males)	N = 15 (12 males and 3 females)	NA
**Subject characteristics**	Male patients performed transurethral resection of the prostate	Patients undergoing thoracic surgery for pneumonectomy	Healthy subjects
**Age (years)**	Ampicillin/sulbactam 1.5g group: 71.4 ± 6.1<br>Ampicillin/sulbactam 3.0g group: 73.2 ± 5.2	59.5 (Mean value)	Males: 25-35
**Body weight (kg)**	Ampicillin/sulbactam 1.5g group: 62.1 ± 9.0<br>Ampicillin/sulbactam 3.0g group: 59.7 ± 10.2	72.5 (Mean value)	Males: 71.7 ± 10
**Renal function**	Ampicillin/sulbactam 1.5g group: CL_{cr}[a], 73.3 ± 21.3 (mL/min)<br>Ampicillin/sulbactam 3.0g group: CL_{cr}[a], 68.9 ± 23.2 (mL/min)	Normal renal function<br>(values not shown)	NA
**Hepatic function**	Ampicillin/sulbactam 1.5g group: T-bil[b], 0.70 ± 0.26 (mg/dL)<br>Ampicillin/sulbactam 3.0g group: T-bil[b], 0.68 ± 0.20 (mg/dL)	Normal hepatic function<br>(values not shown)	NA

NA; Not applicable a, CLcr; Creatinine clearance b, T-bil; Total bilirubin
